# Durvalumab with Gemcitabine and Oxaliplatin in Advanced Biliary Tract Cancer

**DOI:** 10.3390/cancers18121901

**Published:** 2026-06-11

**Authors:** Makenna A. Smack, Jane E. Rogers, Lianchun Xiao, Sunyoung S. Lee, Shubham Pant, Ahmed O. Kaseb, Brandon G. Smaglo, Victoria Higbie, Zishou Ian Hu, Amy An, Milind Javle

**Affiliations:** 1 Pharmacy Clinical Programs, The University of Texas MD Anderson Cancer Center, Houston, TX 77030, USA; 2 Department of Biostatistics, The University of Texas MD Anderson Cancer Center, Houston, TX 77030, USA; 3 Department of Gastrointestinal Medical Oncology, The University of Texas MD Anderson Cancer Center, Houston, TX 77030, USA

**Keywords:** cholangiocarcinoma, biotherapy, chemotherapy, gallbladder cancer, biliary cancer, platinum chemotherapy

## Abstract

Background: Gemcitabine, cisplatin and durvalumab or pembrolizumab are standard first-line treatments for advanced or metastatic biliary tract cancer (BTC). Older patients with BTC may be frail or have contraindications to cisplatin. At our institution, oxaliplatin has been used as an alternative to cisplatin. Methods: In this evaluation, we report the safety and efficacy of gemcitabine with oxaliplatin and durvalumab as a first-line treatment of BTC. The primary objective was overall survival (OS). Secondary objectives included time to progression (TTP), disease control rate (DCR), and the incidence of treatment-related toxicities. Results: Twenty-nine patients were included. The majority were Caucasian (97%) and had an Eastern Cooperative Oncology Group (ECOG) performance status of 0–1 (97%). Median age was 72 years old. Sixty-six percent had intrahepatic cholangiocarcinoma. Baseline renal insufficiency and/or hearing impairment were the most common reasons for cisplatin contraindication. Median follow-up was 20.6 months. Treatment cycles were every 28 days with durvalumab (1500 mg) given on day 1 and gemcitabine (range 600 mg/m^2^–1000 mg/m^2^) plus oxaliplatin (median dose 70 mg/m^2^) given on days 1 and 15. Median OS was 15.7 months (95% CI: 6.9-NA), median TTP was 6.7 months (95% CI 3.88-NA), and DCR was 76%. Median time on treatment was 3.15 months. Twelve patients (41%) required a dose adjustment, with myelosuppression as the most common toxicity. Conclusions: Oxaliplatin, in combination with durvalumab and gemcitabine, is a suitable platinum substitute for advanced BTC patients when cisplatin is contraindicated. Our analysis showed similar efficacy and no new safety concerns. Given the small sample size, our analysis is hypothesis-generating and calls for a larger prospective analysis.

## Simple Summary

Gemcitabine and cisplatin plus durvalumab or pembrolizumab are recommended first-line treatments for advanced or metastatic biliary tract cancer. Cisplatin can be contraindicated in certain populations. We explored, retrospectively, the efficacy and safety of an alternative platinum analog, oxaliplatin. In 29 patients, gemcitabine with oxaliplatin and durvalumab as a first-line treatment of biliary tract cancer was well tolerated with similar efficacy reported with front-line cisplatin-based regimens.

## 1. Introduction

Biliary tract cancer (BTC) is a heterogenous group of rare malignancies encompassing intrahepatic, extrahepatic and hilar cholangiocarcinoma, gallbladder cancer and ampullary cancer. In the United States in 2025, there were estimated to be 12,610 new cases with an estimated 4400 deaths from BTC [1]. The majority of BTC diagnoses are made in the advanced stages where curative resection is not possible. For decades, the standard-of-care treatment for BTC was gemcitabine plus cisplatin based on the results of the ABC-02 trial [2]. However, in recent years, based on the results of the TOPAZ-1 and KEYNOTE-966 trials, gemcitabine plus cisplatin with immune checkpoint therapy (durvalumab or pembrolizumab) is now the standard first-line regimen for advanced or unresectable BTC [3,4].

Durvalumab, an anti-programmed death-ligand 1 (PD-L1) agent, with gemcitabine and cisplatin demonstrated a 7.2-month median progression-free survival (PFS) and 12.8-month median overall survival (OS) [3]. Similarly, pembrolizumab, an anti-programmed death-1 (PD-1) agent, with gemcitabine and cisplatin resulted in a 6.5-month PFS and 12.7-month median OS [4]. While PD-L1 expression has become a predictive biomarker in various solid tumors, its use in BTC continues to be limited [5,6]. Currently, the only immunologic markers predictive of durable responses to immunotherapy in BTC are mismatch repair deficiencies (deficient mismatch repair (dMMR) or microsatellite instability-high (MSI-H)) or high tumor mutation burden (TMB-H). TMB-H and dMMR/MSI-H are both rare in BTC, with a reported incidence of <5% of all cases [6]. The TOPAZ-1 and KEYNOTE-966 trials reported PD-L1 status but given the low incidence of MSI-H tumors in the study populations and some missing biomarker data, the authors were unable to assess outcomes by MSI or PD-L1 status, suggesting the reported OS benefits were not attributable to MSI status or PD-L1 expression [3,4].

Gemcitabine has long been the backbone of chemotherapy regimens for the treatment of BTC [2]. Gemcitabine is metabolized to diphosphate and triphosphate nucleosides by nucleoside kinases [7]. Studies suggest that administering gemcitabine at a fixed-dose rate (FDR) of 10 mg/m^2^/min may impact efficacy compared to higher doses given over the standard 30 min infusion times. While numerically higher survival was reported with FDR gemcitabine-based regimens, outcomes did not satisfy criteria for superiority over doses given over 30 min [8,9,10]. FDR gemcitabine safety is comparable to 30 min infusions, but is noted to cause higher rates of grade 3/4 neutropenia and thrombocytopenia [9,10]. While FDR gemcitabine has only been evaluated in pancreatic cancer populations, not BTC exclusively, both tumor types are treated with gemcitabine- and fluoropyrimidine-based regimens and share anatomic origins with overlapping tumor biology. The NCCN Guidelines for Pancreatic Cancer consider FDR gemcitabine to be a reasonable alternative to standard 30 min gemcitabine infusions [8].

Cisplatin is a platinum analog indicated in the treatment of various malignancies. It carries multiple black-box warnings of nephrotoxicity, neurotoxicity, myelosuppression and nausea and vomiting. Pre- and post-treatment with intravenous (IV) fluids is recommended to reduce the risk of nephrotoxicity. The risk of these toxicities may preclude the use of cisplatin in patients with pre-existing renal insufficiency, hearing impairment, poor performance status, or conditions where fluid boluses may cause adverse events (i.e., heart failure) [11]. Additionally, nationwide shortages of platinum analogs, particularly in recent years, have necessitated the development of alternative treatment options when these medications are not available for use [12].

Oxaliplatin is a platinum analog commonly used in combination with fluoropyrimidines or gemcitabine in BTC and various other gastrointestinal malignancies [13,14,15,16]. In gastric cancer, oxaliplatin-based regimens demonstrated similar efficacy with lower rates of toxicity compared to cisplatin-based regimens. Patients treated with cisplatin are reported to experience more nephrotoxicity, nausea and vomiting, whereas neuropathy and hypersensitivity reactions are reported at higher rates with oxaliplatin-based regimens [17]. BTC is most often diagnosed in people aged 65–74 years [1]. Older patients are more likely to have multiple comorbidities, may not be surgical candidates, and can be frail at diagnosis [17]. This can limit their ability to receive standard chemotherapy and immunotherapy for BTC, particularly due to the intensity of adverse effects with cisplatin. At our institution, gemcitabine and cisplatin are often prescribed in an every-two-week schedule with durvalumab given once every four weeks for both tolerability and patient convenience. In light of recent cisplatin shortages, and often based on patient-specific factors, oxaliplatin is selected as a substitute instead of cisplatin with gemcitabine and durvalumab. The purpose of this retrospective analysis was to review the safety and efficacy of every-two-week gemcitabine and oxaliplatin (GemOx) with every-four-week durvalumab in advanced or metastatic BTC patients.

## 2. Materials and Methods

We performed a retrospective review of patients with advanced or metastatic BTC who were treated with GemOx and durvalumab from 30 September 2022 to 14 January 2026 at UT MD Anderson (MDA). The primary outcome was overall survival (OS), defined as the treatment start date to date of death or last follow-up for patients living at the time of data collection. Categorical data were tabulated with frequency and percentage; continuous data were summarized using descriptive statistics. The Kaplan–Meier method was applied to estimate time to event outcomes.

Eligible patients were adults with confirmed advanced or metastatic BTC who received GemOx and durvalumab as front-line therapy at MDA. Notably, patients with recurrent disease were evaluated. Patients must have received this treatment at our center along with radiographic follow-up every 8–12 weeks at our center. Any patients who received recommendations for GemOx and durvalumab but received treatment elsewhere were excluded. Secondary outcomes included time to progression (TTP) defined from treatment start date to treatment end date and disease control rate (DCR) at the first radiographic evaluation. TTP was also noted with only disease progression as an event; all patients without progression were censored at the end treatment date. We utilized TTP given the retrospective nature of our review. TTP allows us to focus specifically on outcomes pertaining to the drug therapy, capture time on treatment via discreet data points, and avoid capturing mortality from other causes. Disease control was classified as any disease response or stability per radiology review. Toxicities were evaluated based on the need for dose reductions or therapy discontinuation. Time on treatment was evaluated based on the start and end date of treatment doses. Any patients noted to still be on treatment at the time of this review were censored at the date of last follow-up. A landmark time analysis of patients who remained on first-line treatment at 3 months was completed to account for time bias and treatment switching effects. To further evaluate the efficacy of oxaliplatin in comparison to cisplatin-based regimens, a 1:2 matched comparator cohort of patients treated with gemcitabine, cisplatin and durvalumab between 19 February 2018 and 8 March 2024 was utilized.

Data collection points included patient demographics (age, gender, race, ECOG performance status), site of disease (intrahepatic, extrahepatic, hilar and gallbladder), baseline carbohydrate antigen 19-9 (CA 19-9), relevant molecular testing results, virology status, type of second-line treatment, and date of death or last follow-up. In regard to GemOx and durvalumab treatment, we collected treatment starting date, starting doses and chemotherapy schedule. Date of progression, treatment discontinuation, date of death or last follow-up, and reasons for dose adjustments or dose delays were also included. For the matched comparator cohort, patients were matched 1:2 on age, sex, race, tumor stage, and ECOG performance status. The outcome measure for the matched cohort was median overall survival (OS). Our study was approved by the Institutional Review Board and a waiver of consent was granted due to patients being lost to follow-up, no longer at the institution, or having died.

## 3. Results

Twenty-nine patients were included with a median age of 72 years old (range 56–85 years old). Patients were mostly Caucasian (97%) and had an ECOG performance status of 0–1 (97%). Slightly more male patients (55%) were in the analysis. Sixty-six percent had intrahepatic cholangiocarcinoma, 3% extrahepatic cholangiocarcinoma, 14% hilar cholangiocarcinoma, and 17% gallbladder cancer. Ninety-three percent had locally advanced or metastatic disease. No patients had MSI-H or dMMR disease. PD-L1 status was not checked in the majority of cases. Only one patient had PD-L1-positive disease (PDL combined positive score > 1). Tissue molecular analysis was performed on tissue when available (90%). Fourteen patients had no alterations or mutations. Four patients (13%) had a fibroblast growth factor receptor (FGFR) mutation or fusion. Three patients (10%) had Kristen rat sarcoma (KRAS) mutations (G12D, G12C, and G12A). Two patients (7%) had an isocitrate dehydrogenase 1 (IDH1) mutation. One patient (3%) had a germline BRCA 2 mutation. Ninety percent had no hepatitis viral history. Seventy-nine percent (*n* = 23) had no previous history of prior therapy. Overall demographics are included in Table 1.

Reasons patients did not receive cisplatin could be either singular or multifactorial (Table 1). The most common reason was due to baseline renal insufficiency (*n* = 15, 52%). Cisplatin use was also avoided in those with baseline hearing impairment (*n* = 8, 28%). Additional reasons were due to cisplatin national shortage (*n* = 2), previous reaction or intolerance to cisplatin or carboplatin (*n* = 2), or functional status concerns/age (*n* = 3).

**Figure 1:**
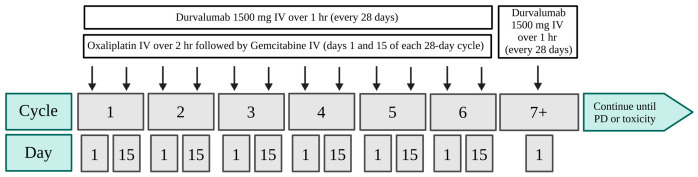
Treatment schedule. Hour (hr), milligram (mg), intravenous (IV), progression of disease (PD). Reference Table 2 for starting doses of oxaliplatin and gemcitabine and gemcitabine infusion rates.

**Figure 2:**
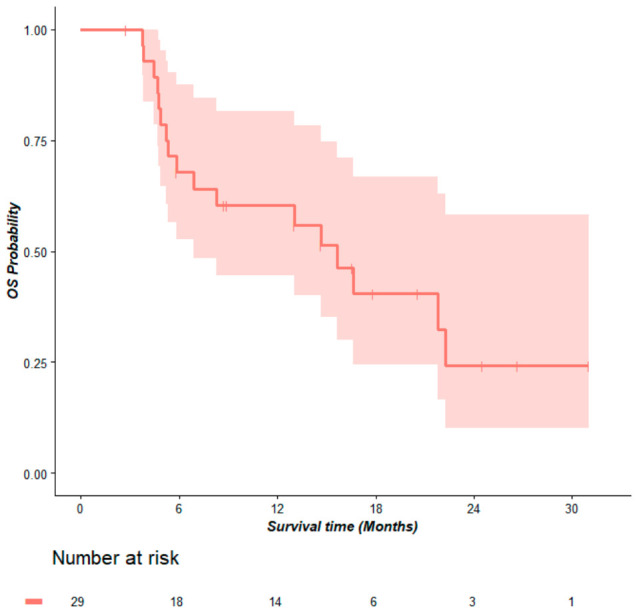
Overall survival. Shaded area represents the 95% confidence interval of the survival probability.

**Figure 3:**
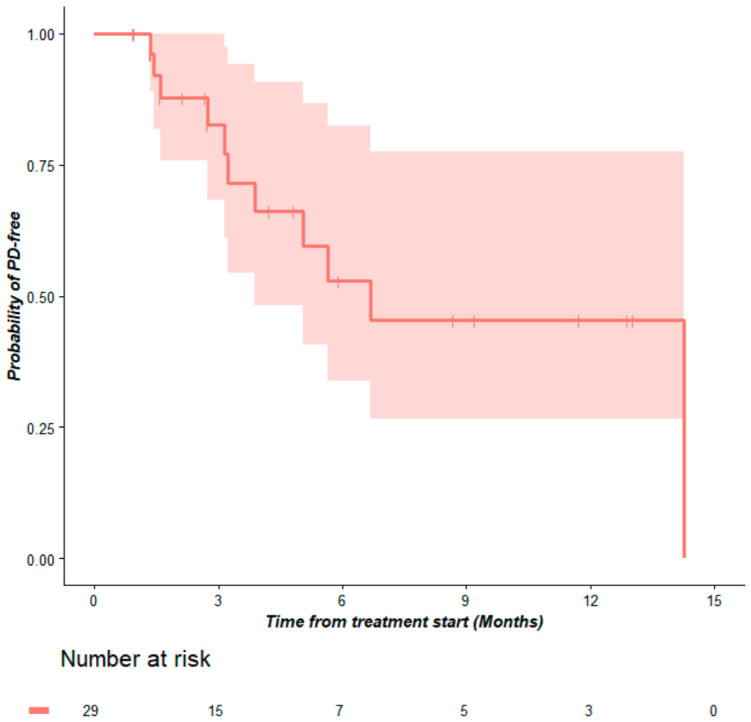
Time to progression. Shaded area represents the 95% confidence interval of progression-free probability.

Median follow-up was 20.6 months (range 95% CI 14.7-NA). Cycle length was every 28 days with gemcitabine and oxaliplatin given on days 1 and 15, and durvalumab given on day 1 (Figure 1). Durvalumab was dosed at 1500 mg flat dose. Gemcitabine was given at an FDR (10 mg/m^2^/minute) in 13 patients (45%) with the remainder receiving gemcitabine at a standard administration rate of 30 min. All those that received FDR gemcitabine had a starting dose of 600 mg/m^2^. Those with standard-rate gemcitabine had a median starting dose of 900 mg/m^2^ (range 600 mg/m^2^–1000 mg/m^2^). Median oxaliplatin starting dose was 70 mg/m^2^ (range 42.5 mg/m^2^–85 mg/m^2^). Full dosing specifics are included in Table 2. Oxaliplatin was given for a median of 2.5 months (range 1 month–7 months) while gemcitabine was given for a median of 3.25 months (range 1.5 months–13 months). Durvalumab was given for a median of 3 months (range 2 to 14 months). Mean total time on treatment for all patients was 4.69 months (range 0.95–14.26 months). Three patients transitioned to receive maintenance treatment with durvalumab (*n* = 1) or durvalumab and gemcitabine (*n* = 2). At the data cutoff for this review, two patients were still receiving treatment. Median OS was 15.7 months (95% CI: 6.9-NA) (Figure 2). One-year survival rate was 60.3%. Median TTP disease was 6.7 months (range 95% CI: 3.88-NA) (Figure 3). DCR was 76% (95% CI: 57.9–87.8%). Outcomes are summarized in Table 3. At 3 months, there were 15 patients who survived at least 3 months and remained on first-line GemOx and durvalumab. Of these 15 patients, 9 died. The median OS time was 13.6 months (95% CI: 5.28, NA). The 12-month survival probability was 58.3% (95% CI: 37.4%, 90.9%). The number of patients who survived at least 6 months and were still on first-line GemOx and durvalumab at 6 months was seven. Landmark analysis at 6 months was not completed due to the limited sample size.

Sixty consecutive intrahepatic cholangiocarcinoma patients who received gemcitabine + cisplatin + durvalumab during a similar time period were included in the matched control. Median age was 66 (range 41–83 years old). Fifty percent were male and fifty percent female. Most patients were Caucasian (83%) and had an ECOG performance status of 0–1 (100%). Most patients had metastatic disease (80%). Median follow-up time for this cohort was 42.6 months and median OS was 15.3 months (95% CI 13.3–27.1). Twelve-month survival probability was 66.7%.

Twelve patients (41%) required at least one dose reduction of gemcitabine, oxaliplatin or both medications. Reasons for adjustments could be singular or multifactorial. Reasons for dose adjustments were thrombocytopenia and/or anemia (*n* = 8), neuropathy (*n* = 3), fatigue (*n* = 3), and/or appetite loss (*n* = 2). Thirteen (45%) patients experienced at least one dose delay while on treatment, with only three patients having two or more delays. The most common reasons for treatment delays were anemia, thrombocytopenia, and fatigue. Reasons for discontinuing treatment included disease progression (*n* = 10), transition to local therapy (*n* = 7), declining performance status and/or poor tolerance (*n* = 5), resection (*n* = 2), recurrent infections (*n* = 1), hypersensitivity, (*n* = 1), and death (*n* = 1). This death occurred close to treatment time; however, based on retrospective review, it was not deemed to be due to acute chemotherapy or immunotherapy toxicities. Four patients (13.7%) had one hospital admission while on therapy. Reasons for admission were acute kidney injury (*n* = 1), nausea/vomiting (*n* = 2), and oxaliplatin hypersensitivity (*n* = 1). Most patients received second-line therapy (59%). Second-line therapy consisted of irinotecan/liposomal irinotecan-based therapy, targeted therapy, or a clinical trial.

## 4. Discussion

BTCs represent a rare group of malignancies. Given this rarity, treatment advancements can be challenging due to pharmaceutical interest in rare tumors and the time necessary for trials to accrue. Advancements can be slow as evidenced by the >10 years between ABC-02 and the TOPAZ-1 and KEYNOTE-966 trials for the standard front-line therapy to change [2,3,4]. Oxaliplatin and carboplatin are both well established for having less nephrotoxicity, ototoxicity, and emesis than cisplatin [11,13,17,18]. As BTC patients can be elderly with chronic comorbidities and may already be on other potentially nephrotoxic medications, we must find alternatives for these patients to give them full treatment potential. At our center, when cisplatin is contraindicated or unavailable due to shortages, we utilize oxaliplatin as a substitute. Our review of 29 patients shows the clinical feasibility of utilizing oxaliplatin as an alternative platinum agent in advanced BTC patients who have contraindications to cisplatin-based therapy. Our results of median OS of 15.7 months, median TTP of 6.7 months, and DCR of 75.9% showed similar efficacy to utilizing a different platinum agent. TOPAZ-1 and KEYNOTE-966 reported median OS of ~13 months with median PFS of ~7 months [3,4]. Additionally, treatment was well tolerated with only 41% of patients requiring dose modifications with no unforeseen adverse effects reported. Any-grade toxicity in TOPAZ-1 was reported at 99.4%. Admissions were seen in 13.7% of patients on durvalumab plus GemOx, similar to the serious toxicity noted in TOPAZ-1 (15.7%). Seventeen percent of patients discontinued treatment due to tolerance/declining performance status, similar to the discontinuation rate of 13% in the TOPAZ-1 study. In addition to aligning with the median OS seen in both TOPAZ-1 and KEYNOTE-966, our institutional 1:2 matched cohort of intrahepatic cholangiocarcinoma patients treated with gemcitabine + cisplatin + durvalumab showed a similar median 15-month OS.

The National Comprehensive Cancer Network (NCCN) lists GemOx as an alternative recommended regimen for primary treatment of unresectable BTC disease [6]. GemOx has been utilized in advanced BTC based on phase 2 evaluations and a phase 3 non-inferiority trial [16,19,20,21]. The results of these trials show similar outcomes to our analysis. This combination has also been evaluated in a phase 3 adjuvant trial owing to its safety profile in this population [22]. Sun et al. reported a phase 3b single-arm, open-label, multicenter Chinese study of GemOx with durvalumab in the front-line advanced BTC setting [23]. Median follow-up at time of reporting was approximately 3 months. Given the short follow-up, efficacy results were not reported, but the authors reported no new safety concerns. No phase 3 trials in advanced BTC are currently evaluating GemOx with immune checkpoint inhibitor therapy. There are, however, combination trials with oxaliplatin as the platinum backbone in phase 2 trials ongoing in BTC [24,25,26,27]. Recently, Shi G.M. and colleagues evaluated gemcitabine, oxaliplatin, toripalimab and lenvatinib in a phase 2–3 trail of neoadjuvant treatment for patients with resectable high-risk intrahepatic cholangiocarcinoma, further demonstrating the activity of oxaliplatin in BTC [14].

Recent developments in BTC treatment have shown the importance of molecular profiling not only for potential actionable mutations but also in regard to prognostic indicators. Our study had 13% FGFR alterations, 10% KRAS mutations, 7% IDH mutations, and 3% BRCA mutations. In the TOPAZ-1 trial, these mutation profiles slightly differed (3% FGFR, ~20% KRAS mutations, 9% IDH, and 4% BRCA) [28,29]. FGFR alterations are known to be more favorable [30]. Also known is that KRAS mutations are known to be poorer in prognosis. Therefore, our study had slightly more favorable mutational alterations compared to the TOPAZ-1 trial; however, our incidences align with reports for the overall BTC population [6].

Limitations are present in our review, including the small sample size and the single-center retrospective design. Overall baseline characteristics of our cohort are heterogenous; due to the small sample size, subgroup analyses of the results were not performed. Given known racial and geographic variations in BTC incidence, including higher prevalence among Asian and Hispanic populations, the predominantly Caucasian population in this study may limit global generalizability. Additionally, we were unable to retrospectively evaluate reasons why starting treatment doses were lower as overall the performance status for our cohort was documented as 0–1. Reasons regarding why oxaliplatin was only administered for a median of 2.5 months are multifactorial, including neuropathy, increasing fatigue, anemia and thrombocytopenia. Two dosing schemes were used for gemcitabine (standard 30 min rate and FDR) which could have impacted toxicity patterns as it is known that FDR gemcitabine can cause more grade 3/4 neutropenia and thrombocytopenia, primary reasons for dose reductions in our population. Providers at our institution use both gemcitabine dosing strategies based on various reports utilizing these different strategies [3,7,8,9,15]. Lack of PD-L1 expression data for our population is a limitation, though at present there are no validated predictive biomarkers for utilizing chemotherapy with immune checkpoint inhibitors in advanced or metastatic BTC [10]. Despite these limitations, our findings along with long follow-up on this regimen provide optimism for oxaliplatin as an alternative to cisplatin. Our analysis is hypothesis-generating and calls for prospective, multicenter validation in a larger cohort with prospective biomarker collection.

## 5. Conclusions

Our results provide an alternative to cisplatin-based chemotherapy. Treatment with GemOx plus durvalumab was well tolerated with results aligning with current standard front-line BTC treatment. Given the rarity of BTC, a dedicated, prospective study utilizing an alternative-based regimen is not likely. Therefore, retrospective evaluations including ours and extrapolating from prior literature point to oxaliplatin as a substitute platinum agent. For advanced BTC patients with contraindications to cisplatin therapy, oxaliplatin may be an option in combination with gemcitabine and durvalumab or pembrolizumab.

## Figures and Tables

**Table 1:** Patient demographics and baseline characteristics.

Parameter	Durvalumab Plus Gemcitabine and Oxaliplatin (*n* = 29)
Median age (range), yr	72 (56–85)
Female sex, no (%)	13 (45)
Caucasian, no (%)	28 (97)
ECOG performance status, no (%)	
0	11 (38)
1	17 (59)
2	1 (3)
Primary tumor type, no (%)	
Intrahepatic	19 (66)
Extrahepatic	1 (3)
Hilar	4 (14)
Gallbladder	5 (17)
Disease status, no (%)	
Initially unresectable	23 (80)
Recurrent	6 (20)
Disease classification, no (%)	
Locally advanced	12 (41)
Metastatic	17 (59)
Virology status, no (%)	
No viral hepatitis	26 (90)
Active hepatitis B or active history	1 (3)
Prior hepatitis C	2 (7)
Baseline CA 19-9–no (%)	
<500 Units/mL	21 (72)
≥500 Units/mL	8 (28)
MSI status, no (%)	
High	0
Stable	28 (97)
Missing *	1 (3)
PD-L1 status, no (%)	
Unavailable	28 (97)
CPS > 1 **	1 (3)
Reasons for cisplatin contraindication	
Renal insufficiency	15 (52)
Hearing impairment	8 (28)
Cisplatin shortage	2 (7)
Previous hypersensitivity	2 (7)
Functional status concerns/age	3 (10)

* MSI status missing includes not tested. ** 1 patient reported CPS 22 and was MSI-stable. ECOG denotes Eastern Cooperative Oncology Group, MSI microsatellite instability, PD-L1 programmed cell death ligand 1, CA 19-9 cancer antigen 19-9.

**Table 2:** Treatment starting doses *.

Medication	Median Dose (Range)
Durvalumab (*n* = 29), mg	1500
Gemcitabine FDR (*n* = 13), mg/m^2^	600
Gemcitabine Standard-Dose Rate (*n* = 16), mg/m^2^	900 (600–1000)
Oxaliplatin (*n* = 29), mg/m^2^	70 (65–85)
**Dosing Breakdown per Patient**	**Dose**
Gemcitabine Standard-Dose Rate, mg/m^2^	600 (*n* = 4)
	700 (*n* = 1)
	750 (*n* = 3)
	1000 (*n* = 8)
Oxaliplatin, mg/m^2^	42.5 (*n* = 1)
	60 (*n* = 1)
	65 (*n* = 10)
	70 (*n* = 3)
	75 (*n* = 1)
	85 (*n* = 13)

* Fixed-dose rate (FDR) denotes infusion time of 10 mg/m^2^/minute. Standard-dose rate denotes a 30 min infusion time. Bold format used within table to visually separate two categories of data reported in the table.

**Table 3:** Efficacy outcomes.

Parameter	Durvalumab Plus Gemcitabine and Oxaliplatin (*n* = 29)
Median overall survival (mo)	15.7 (95% CI: 6.9–NA)
Median time to progression (mo)	6.7 mo (95% CI: 3.9–NA)
	
First radiographic scan results *	
Disease control (%)	76 (95% CI: 57.9–87.8)
Progression (%)	24 (95% CI: 12.2–42.1)

* First radiographic scans were completed after 2 months of treatment for all patients. Not available (NA)

## Data Availability

The data presented in this study are available on request from the corresponding author due to privacy reasons.
